# Cryo-EM reconstruction of the Cafeteria roenbergensis virus capsid suggests novel assembly pathway for giant viruses

**DOI:** 10.1038/s41598-017-05824-w

**Published:** 2017-07-14

**Authors:** Chuan Xiao, Matthias G. Fischer, Duer M. Bolotaulo, Nancy Ulloa-Rondeau, Gustavo A. Avila, Curtis A. Suttle

**Affiliations:** 1 Department of Chemistry, University of Texas at El Paso, El Paso, TX 79968 USA; 2 Max Planck Institute for Medical Research, Department of Biomolecular Mechanisms, 69120 Heidelberg, Germany; 3 Departments of Earth, Ocean & Atmospheric Sciences, Microbiology & Immunology, and Botany, and The Institute for the Oceans and Fisheries, University of British Columbia, Vancouver, BC, V6T 1Z4, Canada; Canadian Institute for Advanced Research, Toronto, ON M5G 1M1 Canada

## Abstract

Whereas the protein composition and overall shape of several giant virus capsids have been described, the mechanism by which these large capsids assemble remains enigmatic. Here, we present a reconstruction of the capsid of Cafeteria roenbergensis virus (CroV), one of the largest viruses analyzed by cryo-electron microscopy (cryo-EM) to date. The CroV capsid has a diameter of 3,000 Å and a Triangulation number of 499. Unlike related mimiviruses, the CroV capsid is not decorated with glycosylated surface fibers, but features 30 Å-long surface protrusions that are formed by loops of the major capsid protein. Based on the orientation of capsomers in the cryo-EM reconstruction, we propose that the capsids of CroV and related giant viruses are assembled by a newly conceived assembly pathway that initiates at a five-fold vertex and continuously proceeds outwards in a spiraling fashion.

## Introduction

Viruses with long dsDNA genomes (>200 kilobases) and large particles (>0.2 µm) are a common occurrence in nature and several new families of giant viruses have been reported in recent years^1–6^, which inspired various discussions regarding their evolutionary origin^7–11^. However, whereas rapidly advancing DNA sequencing methods facilitate genomic analysis of giant viruses, structural studies of large viral capsids are lagging behind. In contrast to small and medium-sized capsids, the >200 nm isometric capsids of giant DNA viruses still pose a significant technical challenge for high-resolution methods such as X-ray crystallography and cryo-EM^12^. Other techniques, including atomic force microscopy, scanning electron microscopy, and X-ray free electron lasers, have been used to study giant virus structures, but are unable to provide near-atomic resolution^13–16^. During the last decade, cryo-EM has become an increasingly powerful tool to determine the structure of virus particles, circumventing the need for crystallization^17–20^. The resolution of cryo-EM reconstruction of viruses has gradually improved from sub-nanometer to near atomic levels^21–30^. In addition, combined with X-ray crystallography of purified capsid proteins, it is possible to fit the atomic structures of individual components into the cryo-EM reconstruction map and determine a pseudo-atomic structure^31–34^. Examples for the successful combination of X-ray crystallography and cryo-EM to determine large DNA virus structures are Paramecium bursaria Chlorella virus 1 (PBCV-1)^35, 36^ and Chilo iridescent virus (CIV)^37^.

Here, we push the limits of cryo-EM application to large virus particles by reconstructing the capsid of Cafeteria roenbergensis virus (CroV)^38^. The giant virus CroV infects the widespread marine zooplankter *Cafeteria roenbergensis*, a single-celled eukaryote and ecologically important bacterivore^39^. Phylogenetically, CroV is a distant relative of the giant Acanthamoeba polyphaga mimivirus (APMV)^1^ and the sole member of the genus *Cafeteriavirus* in the family *Mimiviridae*. With a diameter of 3000 Å, the CroV particle is smaller than the 5000 Å wide capsid of APMV^40^ or the 1.5 × 0.5 µm ovoid particles of *Pithovirus sibericum* ^4^, which renders it more easily accessible to cryo-EM. Nevertheless, obtaining a close-to-nanometer cryo-EM resolution for an intact virus capsid of these dimensions is still problematic. The thickness of the vitreous ice that embeds the viral capsid in cryo-EM is one of the most important factors limiting the resolution. Multiple and inelastic scattering of electrons increases as the ice becomes thicker, which reduces the signal-to-noise ratio. Unlike APMV, the CroV capsid is not covered by a dense layer of 125 nm-long surface fibers that would complicate cryo-EM analysis^41^. Thus, CroV is ideally suited to advance the limit of structural studies of giant viruses. Although crystallization is not required for cryo-EM, thousands of homogenous particles need to be imaged and analyzed to achieve a high resolution structure. In this study, we observed that the CroV particles were homogenous and could be averaged to high resolution. Such detailed structural information of an intact giant virus capsid may help to shed light on their assembly mechanism. Based on our cryo-EM reconstruction of the CroV capsid and by comparison with other giant viruses, we propose a new spiral assembly pathway for the formation of large icosahedral virus capsids.

## Results

### Cryo-EM reconstruction and T-number

**Figure 1:**
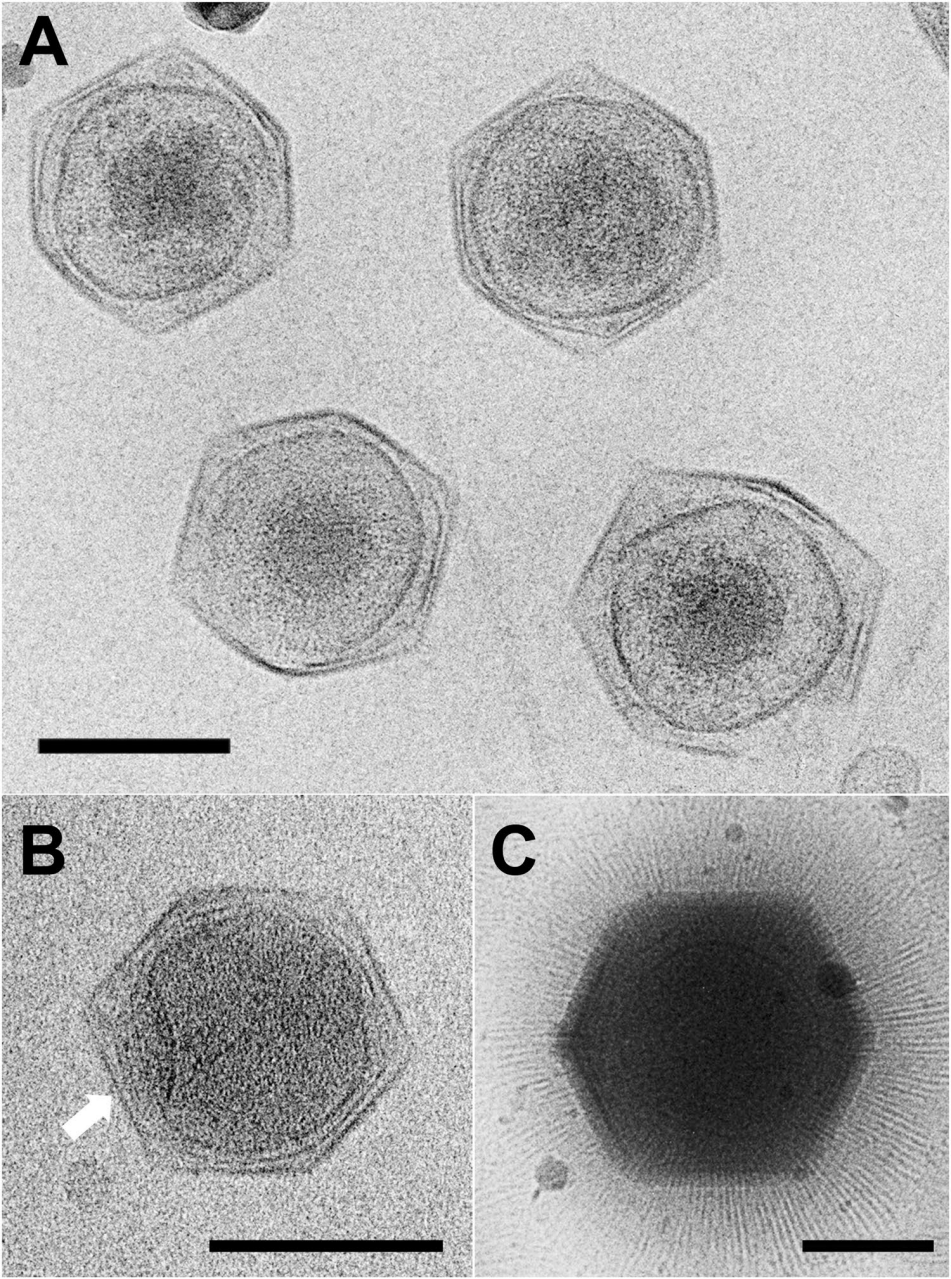
Cryo-EM images of CroV compared to APMV. (**A**) Cryo-electron micrograph of four CroV particles. (**B**) Single CroV particle with concave core depression (white arrow). (**C**) Single APMV particle. Scale bars in (**A**–**C**) represent 2,000 Å.

**Figure 2:**
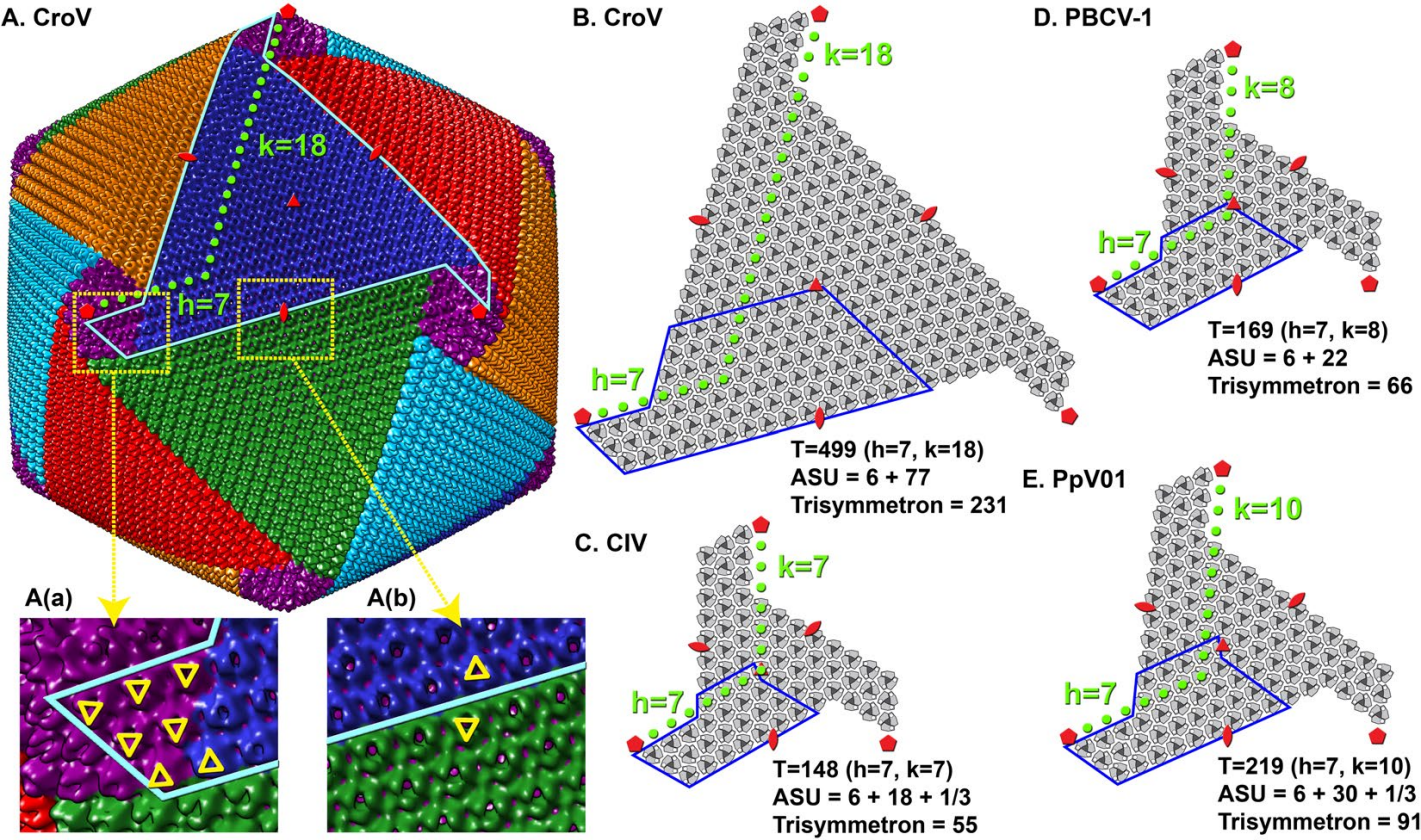
Cryo-EM reconstruction of the CroV virion and capsomer arrangements of other giant icosahedral viruses. (**A**) Reconstruction of the CroV capsid. The isosurface of the map was colored by pentasymmetrons (purple) and trisymmetrons (blue, red, green, cyan and orange). One of the 30 edges of the icosahedron is marked by a cyan line. Two surface areas (a,b) are magnified and selected capsomers are labeled by yellow triangles to show their orientations. (**B**–**E**) Isolated icosahedral faces of CroV, PBCV-1, CIV and PpV01 capsids are shown schematically. Their T-numbers, asymmetric unit capsomer numbers, and trisymmetron capsomer numbers are listed. 5-fold, 3-fold, and 2-fold symbols are indicated in red and ASUs are outlined in blue.

**Figure 3:**
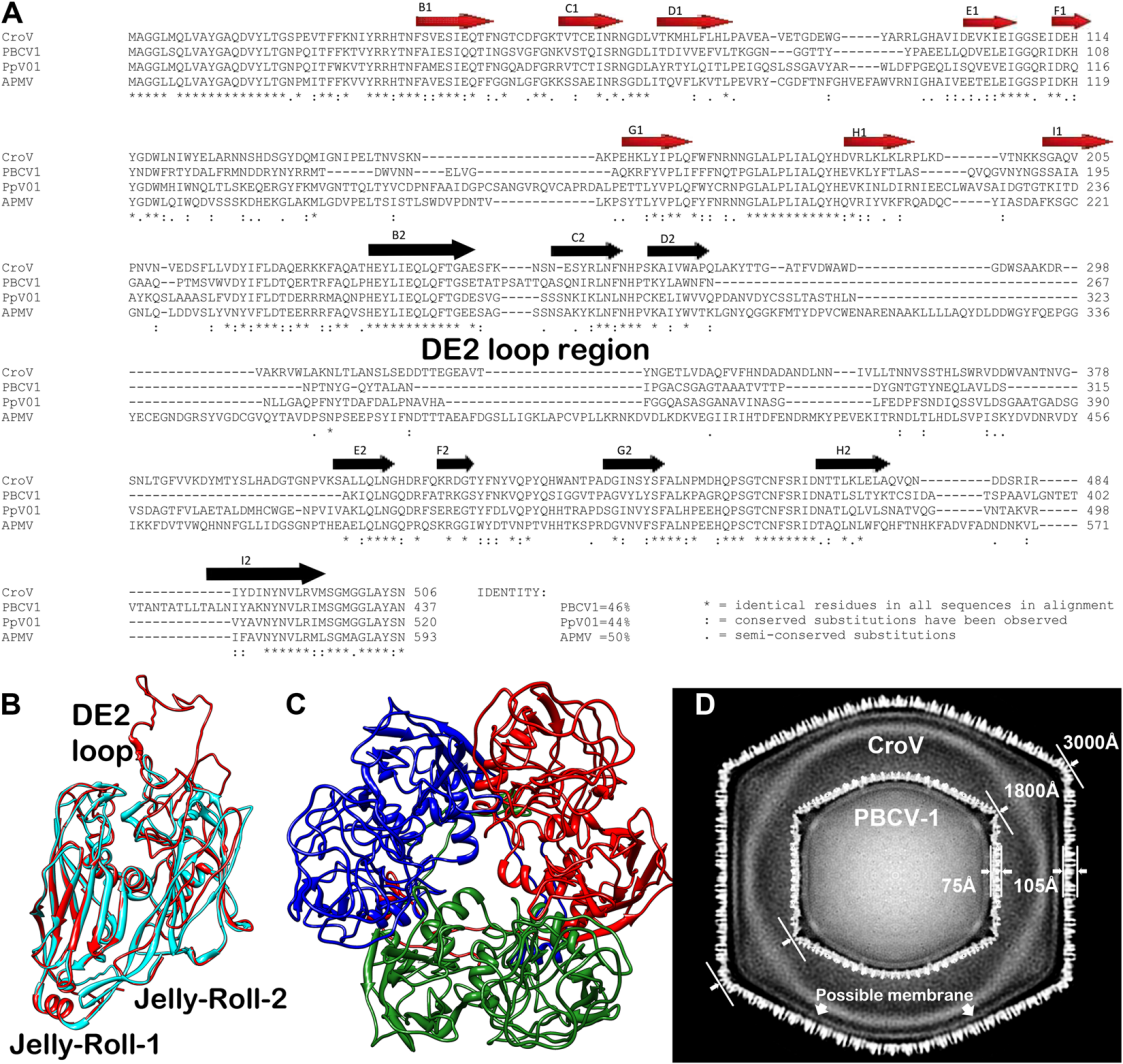
The double jelly-roll major capsid protein (MCP) of large icosahedral viruses. (**A**) Multiple sequence alignment of the MCPs of CroV, PBCV-1, PpV01 and APMV. Both CroV and APMV have large DE2 loops. (**B**) The CroV MCP (red) was homologously modeled and superposed with the X-ray structure of PBCV-1 (cyan) to show the longer DE2 loop of CroV. (**C**) Ribbon diagram of the trimeric homologously modeled CroV double jelly-roll MCP capsomer displaying a pseudo-hexagonal shape. Individual double jelly-roll MCPs are color-coded. (**D**) Central cross-section of the cryo-EM map of CroV superposed with that of PBCV-1. CroV not only has a larger virion diameter (3000 Å vs 1800 Å), its capsid layer is also thicker (105 Å vs 75 Å), which is consistent with the extended DE2 loop shown in panel (B).

Purified from ≈40 L of infected *Cafeteria roenbergensis* culture, we obtained enough CroV sample for cryo-EM data collection (Fig. 1A). In total, 6698 particles were processed and 2471 particles were used in the final cryo-EM reconstruction. The refinement process required about three million CPU hours to reach the final reconstruction. The 21 Å resolution cryo-EM reconstruction of CroV reported here clearly shows individual capsomers on the virion surface (Fig. 2A and video in Supplementary s01). The major capsid protein (MCP) that forms the trimeric capsomers in most icosahedral giant viruses consists of a double “jelly-roll” fold^12^. Each jelly-roll is a wedge-shaped structure composed of eight anti-parallel β-strands^42^. The trimeric capsomer has a pseudo-hexagonal shape with six single jelly-rolls contained in three double jelly-roll MCPs. The vertices of the icosahedral particle are occupied by pentameric capsomers that probably consist of single jelly-roll proteins^30^. A multiple sequence alignment of several giant virus MCPs shows relatively high similarity in β-strand regions, whereas the inter-strand regions often contain insertions of varying length, such as the DE2 loop of the second jelly-roll (Fig. 3A)^12^. These insertions form protrusions on the exterior of the capsomers, conveying a truly trimeric look (magnified areas in Fig. 2A(a) and (b)). Capsomer arrangements of icosahedral viruses can be mathematically described by the triangulation number T, as defined by Caspar and Klug^43^. The T-number is a measurement of how many monomers (e.g. jelly-roll domains) exist in one icosahedral asymmetric unit. By tracing the capsomers in the hexagonal array from one 5-fold vertex to the neighboring one along axes h and k, which follow the center of the MCP (Fig. 2A), the T-number can be calculated using the equation: T = h^2^ + hk + k^2^. Based on the well resolved individual capsomers in our cryo-EM reconstruction, the T-number of the CroV capsid equals 499 (h = 7, k = 18) (Fig. 2B). Previously, the highest accurately determined T-number was that of Faustovirus (T = 277)^44^. The giant APMV capsid is estimated to have a T-number of ≈1000, but so far, technical barriers have prevented high-resolution reconstructions of APMV^41^.

### Surface landscape

Giant virus capsids differ markedly in their surface architecture. APMV has a dense layer of fibers on its surface (Fig. 1C)^45^. The CIV virion has short fibers on most capsomers^37^, while PBCV-1 and Phaeocystis pouchetti virus (PpV01) capsids have fibers only on certain capsomers^36, 46^. In contrast, the CroV capsid is not decorated with fibers (Fig. 1A and B), but has surface protrusions that are almost 30 Å higher than those of PBCV-1 (Fig. 3D), owing to the 83 amino acids longer DE2 loop in the CroV MCP (Fig. 3A and B). This longer loop might play a role in virus-host interactions similar to the fibers of other giant viruses. The absence of external fibers, which convey great physical stability to APMV virions^13, 41^, leads to a higher signal-to-noise ratio in the CroV cryo-EM data. On the other hand, the exposed capsid surface renders CroV virions quite fragile, thus broken and deformed particles were frequently observed (Fig. 1A).

### Unique Vertex and Genome-Containing Core

Many bacteriophages have unique portal structures for DNA packaging^47^ or genome delivery^48^. A so-called “star-gate” portal was discovered on the APMV capsid using transmission and scanning electron microscopy^14^, which was later confirmed by cryo-EM reconstruction with 5-fold averaging^41^. Using the same technique, the PBCV-1 capsid was found to have a modified vertex with a pocket underneath a needle-like spike structure, which may be involved in penetrating the host cell^36^. We also applied 5-fold averaging to the CroV reconstruction in order to detect potential unique vertex modifications. This resulted in densities at the outside of two opposing vertices along the 5-fold averaging axis, which might indicate a unique portal on the CroV capsid. However, due to noise in the data and the limited number of analyzable cryo-EM images, we were unable to confirm the existence of a CroV portal. If such a unique structure exists, it is probably quite small, as we did not observe any obvious portals in the original cryo-EM imagery.

The genome of APMV and related giant viruses is contained in a spherical inner compartment called the viral core, which is separated from the capsid layer by the inner viral membrane at a capsid – core distance of 300–500 Å^41^. The APMV core was found to have a concave depression beneath the unique vertex. Re-examination of the original APMV cryo-EM micrographs showed that the core depression is directly visible for most of the particles. For example, four of the seven particles published in the first cryo-EM study of APMV display this feature^45^. With CroV, we only observed very few particles that had a similarly deformed core (Fig. 1B). Most CroV cores seem to have a more or less spherical shape with a higher electron density in the center (Fig. 1A). In agreement with observations from other icosahedral giant viruses, the CroV cryo-EM reconstruction shows a possible membrane layer just beneath the protein capsid (Fig. 3D). No additional structural layers were observed, as seen in initial cryo-EM studies of APMV^41, 45^.

## Discussion

**Figure 4:**
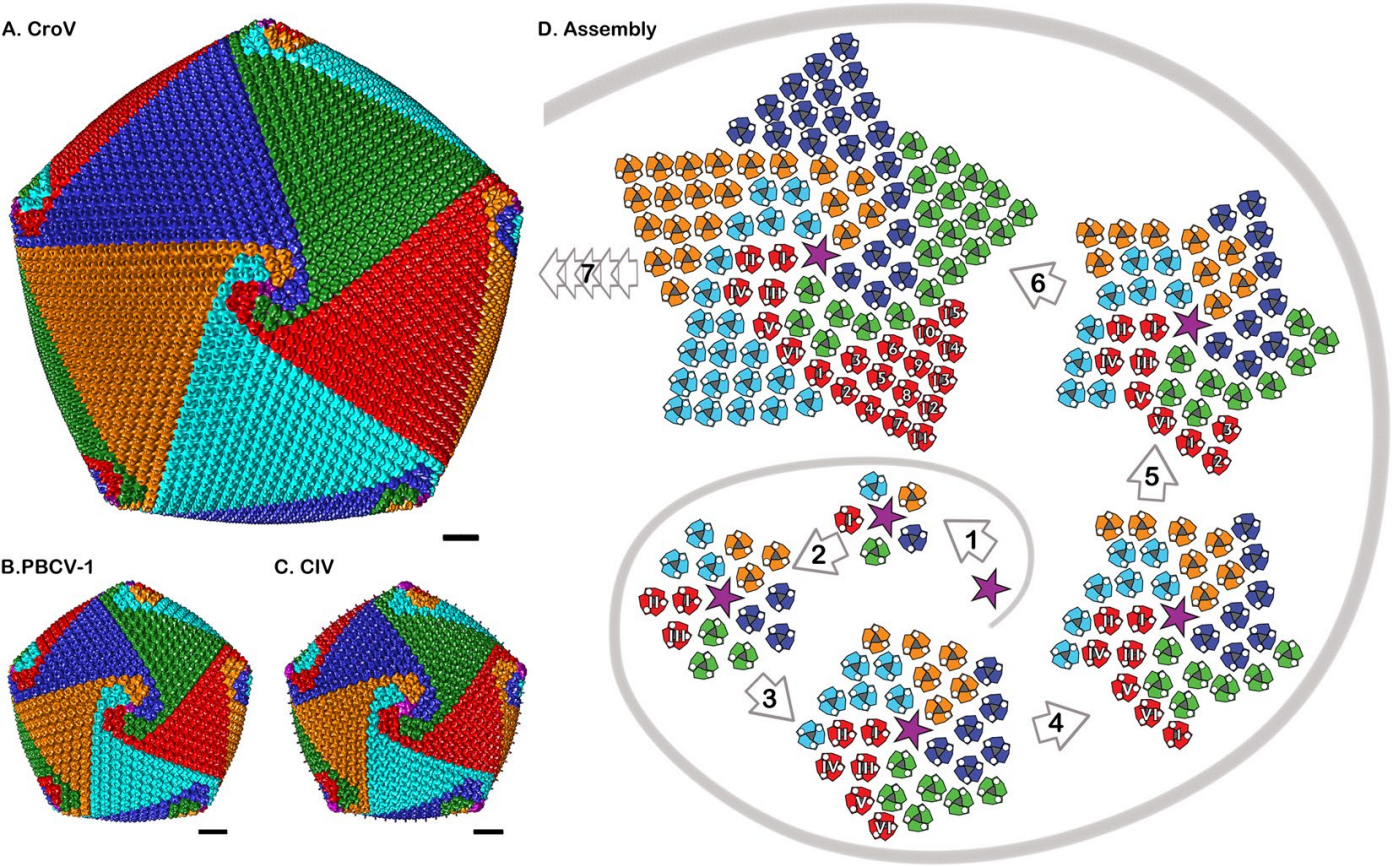
The proposed spiral assembly pathway of large icosahedral capsids. Isosurfaces of CroV (**A**), PBCV-1 (**B**), and CIV (**C**) cryo-EM maps were centered on the 5-fold axis. Capsomers in all three panels are colored based on their orientation in red, blue, green, cyan, and orange. The pentameric capsomers are depicted as purple stars. (**D**) Schematic diagram showing the right-handed spiral assembly pathway following arrows (1) to (6), with multiple extensions (7) of the trisymmetrons to form similar patterns as shown in (**A**–**C**). One set of capsomers (red) is labeled by Roman numerals if they are part of the pentasymmetron and by Arabic numerals if they are part of a trisymmetron.

Large, well-organized triangular arrays of capsomers were first discovered in samples of degraded Sericesthis iridescent virus^49^, which indicated that the capsid of large icosahedral viruses might be assembled from a pre-formed array of MCPs. The 5-fold and 3-fold arrays were named pentasymmetrons and trisymmetrons, respectively. In cryo-EM reconstructions of sufficiently high resolution, the symmetrons can be distinguished from each other by discontinuous lines separating capsomers of different orientations. This distinction is possible because the capsomers have only quasi-six-fold symmetry, but a true three-fold symmetric appearance^12^. The 231 capsomers of one CroV trisymmetron are rotated by 60° compared to the capsomers of a neighboring trisymmetron (Fig. 2A(b)), which clearly defines the trisymmetron boundary. In all available cryo-EM reconstructions of icosahedral giant viruses, including CroV, the pentasymmetrons consistently contain 31 capsomers with three concentric layers of pseudo-hexameric capsomers and one pentameric capsomer at the 5-fold vertex. This arrangement leads to a fixed h-number of 7 for all these giant viruses (Fig. 2B–E). The identical capsomer arrangement around the 5-fold vertices present in CroV, PBCV-1, PpV01, and CIV, leads us to conclude that they are likely assembled in a similar manner, starting with the pentameric capsomer. Analysis of the orientation of pentasymmetron capsomers shows that one of the six capsomers in the asymmetric unit has a different orientation, compared to the other five (Fig. 2A(a)). This unique capsomer is oriented the same way as the capsomer at the tip of a nearby trisymmetron, whereas the other five capsomers are rotated by 60°, creating an obvious boundary between pentasymmetron and trisymmetron. Coloring the capsomers according to their orientation reveals a spiral pattern around the 5-fold vertex that resembles five interlocked golf club heads surrounding the pentameric capsomer (Fig. 4). This is not only true for CroV, but also for PBCV-1 and CIV (Fig. 4B and C). Cryo-tomography studies have shown that the *in vivo* assembly of APMV capsids starts from the 5-fold vertex and proceeds gradually to complete the capsid shell^14, 50, 51^. No pre-assembled arrays or symmetrons have been observed inside the cell. These results challenge the idea that capsids assemble via pre-formed multicapsomeric units^49^. Therefore, we propose a continuous assembly pathway (Fig. 4D and animation in Supplementary s02) based on the common spiral pattern of capsomer orientations observed in icosahedral giant viruses (Fig. 4A–C). According to our model, capsomers first assemble in two layers around the pentameric capsomer, forming five triangles each with the same orientation (Fig. 4D step 1 and 2). For the third layer, instead of extending the triangle, one capsomer will “spiral” into the counterclockwise neighboring triangle (Fig. 4D step 3 and 4). This differently orientated capsomer will then seed the trisymmetron by recruiting more capsomers of the same orientation (Fig. 4D step 5, 6 and 7).

In our model, we considered only the main structural components of the capsid, the MCP and the penton protein. However, the CroV genome encodes three additional paralogous capsid genes that could also influence the assembly process. A proteome analysis of purified CroV particles revealed that all four capsid proteins are present in the virion, although in different quantities^52^. Whereas the MCP was the most abundant protein, it is estimated that the CroV capsid contains only 60 copies of capsid protein 2, and 1–2 copies of the remaining two capsid proteins. Based on these copy numbers, it is possible that capsid protein 2 could be involved in the proposed spiral assembly process. As stated above, one capsomer in the pentasymmetron asymmetric unit is oriented differently (Fig. 2A(a) and capsomer VI in Fig. 4D) and “spirals” into the neighboring trisymmetron. Each pentasymmetron has five of those unique capsomers and there are 60 such unique positions per virion. Hence, the unique pentasymmetron capsomers could be heterotrimers consisting of one capsid protein 2 molecule and two copies of the MCP. The rare capsid proteins 3 and 4 could be involved in the aforementioned unique vertex structure that possibly decorates the CroV capsid. However, the resolution of our current reconstruction does not provide sufficiently detailed structural information to distinguish between different capsid proteins.

In summary, the 21 Å resolution cryo-EM reconstruction of the CroV virion allowed us to accurately determine the structure of a giant virus capsid, revealing the largest T-number reported to date (499), and the biggest trisymmetron consisting of 231 capsomers. Based on the orientation of capsomers around 5-fold axes, we propose that the capsids of CroV and related icosahedral giant viruses are assembled in a spiral mechanism, rather than from preformed MCP arrays, as has been generally accepted for giant icosahedral viruses. It is noteworthy that our assembly model is based on the observation that MCP capsomers are oriented in an identical fashion around the pentasymmetron in several giant icosahedral viruses. Although our model lacks direct experimental support, it is consistent with the continuous capsid assembly observed in cryo-tomography studies^14, 50, 51^. Furthermore, our model focuses only on the initial assembly steps, but does not address the question how the assembly continues to incorporate neighboring pentasymmetrons into the growing capsid, or how capsid size is regulated in giant icosahedral viruses. We hope that future studies will provide experimental data to test our spiral assembly hypothesis and clarify how trisymmetron size and spiral assembly at neighboring 5-fold vertices are controlled.

## Methods

CroV was grown and purified as previously described^52^. CroV samples were loaded on Quantifoil S7/2 grids (Quantifoil Micro Tools GmbH, Germany), blotted manually and frozen using a guillotine style plunging device. The cryo-EM images were recorded on Kodak SO-163 film in a FEI CM300 field emission gun microscope at a calibrated magnification of 20,629 and a dose level of approximately 25 e-/Å^2^ at the Purdue University Cryo-EM facility. Micrographs were scanned on a Nikon Coolscan 9000 with a final pixel size of 6.156 Å. The cryo-EM reconstruction was calculated using the program FREALIGN^53^. Most computation was performed at Texas Advanced Computing Center (TACC). Resolution of the reconstruction was determined using Fourier Shell correlation (FSC) for the capsid part with threshold of 0.333.

### Data Availability Statement

All data associated with the manuscript are available to readers on request.

## Electronic supplementary material

Supplementary s01

Supplementary s02
